# Long-Term Efficacy and Safety of Brigatinib in Crizotinib-Refractory *ALK*+ NSCLC: Final Results of the Phase 1/2 and Randomized Phase 2 (ALTA) Trials

**DOI:** 10.1016/j.jtocrr.2022.100385

**Published:** 2022-07-31

**Authors:** Scott N. Gettinger, Rudolf M. Huber, Dong-Wan Kim, Lyudmila Bazhenova, Karin Holmskov Hansen, Marcello Tiseo, Corey J. Langer, Luis G. Paz-Ares Rodríguez, Howard L. West, Karen L. Reckamp, Glen J. Weiss, Egbert F. Smit, Maximilian J. Hochmair, Sang-We Kim, Myung-Ju Ahn, Edward S. Kim, Harry J.M. Groen, Joanna Pye, Yuyin Liu, Pingkuan Zhang, Florin Vranceanu, D. Ross Camidge

**Affiliations:** a Yale Cancer Center, Yale-New Haven Hospital, New Haven, Connecticut; b Thoracic Oncology Centre Munich, University Hospital of Munich, member of the German Center for Lung Research (DZL, CPC-M), Munich, Germany; c Department of Internal Medicine, Seoul National University College of Medicine and Seoul National University Hospital, Seoul, South Korea; d University of California San Diego Moores Cancer Center, La Jolla, California; e Department of Clinical Oncology, Odense University Hospital, Odense, Denmark; f Department of Medicine and Surgery, University of Parma, Parma, Italy; g University of Pennsylvania Abramson Cancer Center, Philadelphia, Pennsylvania; h Medical Oncology Department, Hospital Universitario, Madrid, Spain; i City of Hope Comprehensive Cancer Center, Duarte, California; j Current Affiliation: Samuel Oschin Cancer Center, Cedars-Sinai Medical Center, Los Angeles, California; k MiRanostics Consulting, Oro Valley, Arizona; l Thoracic Oncology Service, Netherlands Cancer Institute, Amsterdam, The Netherlands; m Karl Landsteiner Institute of Lung Research and Pulmonary Oncology, Department of Respiratory and Critical Care Medicine, Klinik Floridsdorf, Vienna, Austria; n Department of Oncology, Asan Medical Center, Seoul, South Korea; o Division of Hematology-Oncology, Samsung Medical Center, Sungkyunkwan University School of Medicine, Seoul, South Korea; p Department of Pulmonary Diseases, University of Groningen and University Medical Center Groningen, Groningen, The Netherlands; q Oncology Statistics, Takeda Development Center Americas, Inc., Lexington, Massachusetts; r Clinical Science, Takeda Development Center Americas, Inc., Lexington, Massachusetts; s Division of Medical Oncology, Department of Medicine, University of Colorado Cancer Center, Aurora, Colorado

**Keywords:** Anaplastic lymphoma kinase, ALK tyrosine kinase inhibitor, Brigatinib, Crizotinib, Non–small-cell lung cancer

## Abstract

**Introduction:**

We report brigatinib long-term efficacy and safety from phase 1/2 and phase 2 (ALTA) trials in *ALK*–rearrangement positive (*ALK*+) NSCLC.

**Methods:**

The phase 1/2 study evaluated brigatinib 30 to 300 mg/d in patients with advanced malignancies. ALTA randomized patients with crizotinib-refractory *ALK+* NSCLC to brigatinib 90 mg once daily (arm A) or 180 mg once daily (7-d lead-in at 90 mg; arm B).

**Results:**

In the phase 1/2 study, 79 of 137 brigatinib-treated patients had *ALK+* NSCLC; 71 were crizotinib pretreated. ALTA randomized 222 patients (n = 112 in arm A; n = 110 in arm B). Median follow-up at phase 1/2 study end (≈5.6 y after last patient enrolled) was 27.7 months; at ALTA study end (≈4.4 y after last patient enrolled), 19.6 months (A) and 28.3 months (B). Among patients with *ALK*+ NSCLC in the phase 1/2 study, median investigator-assessed progression-free survival (PFS) was 14.5 months (95% confidence interval [CI]: 10.8–21.2); median overall survival was 47.6 months (28.6–not reached). In ALTA, median investigator-assessed PFS was 9.2 months (7.4–11.1) in arm A and 15.6 months (11.1–18.5) in arm B; median independent review committee (IRC)-assessed PFS was 9.9 (7.4–12.8) and 16.7 (11.6–21.4) months, respectively; median overall survival was 25.9 (18.2–45.8) and 40.6 (32.5–not reached) months, respectively. Median intracranial PFS for patients with any brain metastases was 12.8 (9.2–18.4) months in arm A and 18.4 (12.6–23.9) months in arm B. No new safety signals were identified versus previous analyses.

**Conclusions:**

Brigatinib exhibited sustained long-term activity and PFS with manageable safety in patients with crizotinib-refractory *ALK+* NSCLC.

## Introduction

*ALK* gene rearrangements are detectable in approximately 3% to 5% of patients with NSCLC.^1^, ^2^, ^3^ Treatment with ALK inhibitors is the preferred initial systemic approach for *ALK* rearrangement-positive (*ALK*+) metastatic NSCLC.^4^ Crizotinib was the first ALK inhibitor approved by the U.S. Food and Drug Administration (FDA) for the treatment of patients with previously untreated metastatic *ALK*+ NSCLC. Although crizotinib provides improved efficacy and tolerability compared with chemotherapy, most patients experience disease progression on crizotinib within a year.^5^^,^^6^ The central nervous system (CNS) is often the first site of disease progression on crizotinib, reflecting inadequate drug penetration into the brain.^7^, ^8^, ^9^ Other mechanisms of resistance to crizotinib include the acquisition of secondary mutations in *ALK* that interfere with crizotinib binding, amplification of the *ALK* fusion gene, and up-regulation of bypass signaling pathways.^10^ Several next-generation *ALK* inhibitors, including alectinib, ceritinib, brigatinib, and lorlatinib, with activity against mechanisms of resistance to crizotinib, have since been developed and approved for use in *ALK* inhibitor-naive and -resistant NSCLC. Brigatinib first gained approval in 2017 for use in patients with *ALK+* NSCLC with disease progression on or intolerance to crizotinib. In 2020, brigatinib was granted full FDA approval for treatment of *ALK+* NSCLC on the basis of efficacy and safety results from ALTA-1L, a global randomized phase 3 study comparing brigatinib with crizotinib in patients with tyrosine kinase inhibitor (TKI)-naive *ALK*+ NSCLC.^11^

Brigatinib is a next-generation ALK TKI designed to have potent and broad activity against *ALK*-positive rearrangements and a range of *ALK* resistance mutations.^12^, ^13^, ^14^ The recommended dose of brigatinib (180 mg once daily with 7-d lead-in at 90 mg once daily) was established in a multinational phase 1/2 study^15^ and confirmed in the phase 2 ALTA (ALK in Lung Cancer Trial of AP26113) trial in crizotinib-refractory patients with *ALK*+ NSCLC.^16^^,^^17^ Results of interim analyses of each study were previously reported,^15^, ^16^, ^17^ revealing high overall and intracranial objective response rates (ORRs) and durable responses with an acceptable safety profile.

Here, we report long-term efficacy and safety results from the final analyses of the phase 1/2 and phase 2 (ALTA) trials of brigatinib, completed more than 5 years after the last patient enrolled in the phase 1/2 study and more than 4 years after the last patient enrolled in the ALTA trial.

## Materials and Methods

### Study Design and Patients

#### Phase 1/2 Study

The phase 1/2 single-arm, open-label trial (ClinicalTrials.gov identifier: NCT01449461) was conducted in the USA and Spain. The methods, the complete protocol, and eligibility criteria have been published previously.^15^ The dose-escalation phase (phase 1) enrolled patients with histologically confirmed advanced malignancies other than leukemia. The expansion phase (phase 2) enrolled patients with *ALK*+ or *EGFR* T790M-positive NSCLC or other cancers with *ALK* or *ROS1* mutations. Herein, we report long-term outcomes for all patients with *ALK*+ NSCLC treated with brigatinib in any part of the study. In the dose-escalation stage, patients received oral brigatinib at total daily doses of 30 to 300 mg; in the expansion stage, three once-daily oral dosing regimens were assessed: 90 mg once daily, 180 mg once daily, and 180 mg with 7-day lead-in at 90 mg. Results revealed that treatment with brigatinib 180 mg once daily with a 7-day lead-in at 90 mg provided increased benefit, while reducing the incidence of early onset pneumonitis and other pulmonary adverse events (AEs) that had been reported in a subset of patients in the dose-escalation and early expansion phases of the phase 1/2 study.^15^

#### ALTA

The phase 2 ALTA trial (ClinicalTrials.gov identifier: NCT02094573) was an open-label, randomized, multicenter, international study. Methods and the complete study protocol and eligibility criteria have been published.^16^ Eligible patients (≥18 y of age) had locally advanced or metastatic *ALK*+ NSCLC that had progressed while receiving crizotinib; at least one measurable lesion per Response Evaluation Criteria in Solid Tumors (RECIST) version 1.1^18^; and Eastern Cooperative Oncology Group performance status of 2 or less. Patients were stratified by baseline brain metastases status (yes or no) and best previous response to crizotinib (investigator-assessed complete response [CR] or partial response [PR] versus other or unknown response); they were randomized 1:1 to brigatinib 90 mg once daily (arm A) or to 180 mg once daily with a 7-day lead-in at 90 mg (arm B).

In both trials, patients could continue brigatinib until they experienced disease progression or intolerable toxicity. Treatment could be continued after progression at the investigator’s discretion if there was evidence of clinical benefit. In ALTA, patients in arm A could transition to brigatinib 180 mg once daily after progression at 90 mg once daily.

Each trial was conducted in compliance with the ethical principles of the Declaration of Helsinki, the International Council for Harmonisation guideline for Good Clinical Practice, and all applicable local regulations. All patients provided written informed consent. All protocols were approved by local institutional review boards or ethics committees at each site.

### Assessments

In both studies, disease was assessed according to RECIST version 1.1^18^ at baseline and every 8 weeks during treatment (every 12 weeks after cycle 15 in ALTA) and at the end of treatment. In the phase 1/2 study, disease was assessed by the investigators; in ALTA, disease was assessed by the investigators and an independent review committee (IRC). All PRs and CRs were required to be confirmed at least 4 weeks after the initial response. All patients were followed for survival every 3 months for up to 2 years after the initial dose of brigatinib (phase 1/2) or for 2 years after the last patient was enrolled (ALTA). AEs, including laboratory abnormalities, were categorized using the National Cancer Institute Common Terminology Criteria for Adverse Events, version 4.0.

### Outcomes

#### Phase 1/2 Study

The investigator-assessed ORR per RECIST version 1.1^18^ was the primary outcome for four of the five cohorts of the phase 1/2 expansion phase; the CNS response rate per RECIST version 1.1 was the primary outcome for the cohort of patients with *ALK*+ NSCLC with active, measurable, intracranial CNS metastases at baseline. “Active” was defined as brain metastases without previous radiotherapy or with investigator-assessed progression after previous radiotherapy. “Measurable” was defined as CNS lesions of 10 mm or more. Secondary outcomes for all cohorts included progression-free survival (PFS), time to progression, overall survival, and safety and tolerability.

#### ALTA

The primary end point of ALTA was the confirmed ORR, as assessed by the investigator, per RECIST version 1.1.^18^ Secondary end points included confirmed ORR, as assessed by the central IRC, per RECIST version 1.1; CNS response (in patients with active brain metastases, intracranial ORR was assessed by the investigator and confirmed by IRC per RECIST version 1.1); time to response; duration of response; disease control rate (the percentage of patients with best response of CR, PR, or stable disease, per RECIST version 1.1); PFS; overall survival; and safety and tolerability.

### Statistical Analysis

For the phase 1/2 study, data from all patients with *ALK*+ NSCLC who received brigatinib in any part of the study were pooled and analyzed for efficacy and safety. For ALTA, efficacy was analyzed in the intention-to-treat (ITT) population (all randomized patients) and safety was evaluated in the safety population (all patients who received ≥1 dose of brigatinib). For both studies, the exact binomial method was used to calculate confidence intervals (CIs); 97.5% CIs were estimated for the confirmed ORR in ALTA (primary end point) and 95% CIs were used for the other outcomes. Median values and two-sided 95% CIs for time-to-event (duration of response, PFS, and overall survival) analyses were calculated using Kaplan-Meier (KM) methods. Statistical analyses were performed using SAS software (version 9.4, SAS Institute, Inc., Cary, NC).

## Results

### Patients

#### Phase 1/2 Study

**Figure 1:**
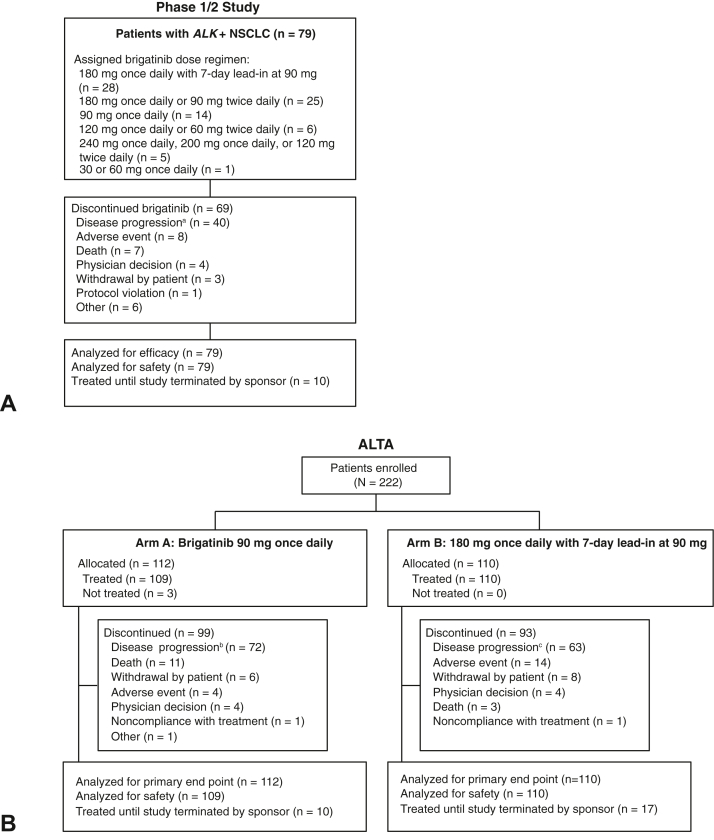
CONSORT diagrams for (*A*) the phase 1/2 study and (*B*) the ALTA trial. ^a^A total of 33 patients had documented disease progression per RECIST version 1.1. Seven patients had clinical disease progression; ^b^A total of 63 patients had documented disease progression per RECIST version 1.1. Nine patients had clinical disease progression; ^c^A total of 50 patients had documented disease progression per RECIST version 1.1. A total of 13 patients had clinical disease progression. *ALK+*, *ALK* rearrangement positive; RECIST, Response Evaluation Criteria in Solid Tumors.

Between September 20, 2011, and July 8, 2014, a total of 137 patients were enrolled in the phase 1/2 study and received brigatinib at doses ranging from 30 mg to 300 mg daily; 79 patients had *ALK*+ NSCLC. Of the patients with *ALK*+ NSCLC, 90% (71 of 79) had previously received crizotinib. Among these 79 patients, the most common brigatinib dosing regimens were 180 mg once daily with 7-day lead-in at 90 mg (n = 28), 180 mg daily (90 mg twice daily or 180 mg once daily; n = 25), and 90 mg once daily (n = 14). The last patient’s final visit on the study was on February 18, 2020, approximately 5.6 years after the last patient was enrolled, with a median follow-up of 27.7 months (range: 0.2–88.3). Median duration of brigatinib exposure in the 79 patients with *ALK*+ NSCLC was 20.0 months (range: 0.03–87.2). There were 10 patients who had no disease progression and were still receiving brigatinib at study end (Fig. 1*A*).

#### ALTA

Between June 4, 2014, and September 21, 2015, a total of 222 patients with crizotinib-refractory *ALK*+ NSCLC were enrolled and allocated to arm A (n = 112) or arm B (n = 110) in ALTA. The last patient’s final visit was February 27, 2020, approximately 4.4 years after the last patient was enrolled. Median follow-up was 19.6 months (range: 0.1–62.8) in arm A and 28.3 months (range: 0.1–66.8) in arm B. Median duration of brigatinib exposure was 13.2 months (range: 0.03–61.8) in arm A and 17.1 months (0.1–66.7) in arm B. At the end of the study, 10 patients in arm A and 17 patients in arm B had no disease progression and were still receiving brigatinib (Fig. 1*B*).

Demographic and clinical characteristics at baseline have been published for both studies.^15^^,^^16^

### Efficacy: Phase 1/2 Study

#### Response Characteristics

Among the 79 patients with *ALK*+ NSCLC in the phase 1/2 study, the confirmed ORR per investigator assessment was 67% (95% CI: 56–77), with median KM-estimated duration of response of 14.9 months (95% CI: 9.9–29.5) (Table 1). In the 28 patients with *ALK*+ NSCLC who received the recommended brigatinib dosing regimen (180 mg once daily with 7-d lead-in at 90 mg), the confirmed ORR was 79% (95% CI: 59–92), with median duration of response of 14.8 months (95% CI: 7.9–33.3). Response rates and characteristics were similar for patients with *ALK*+ NSCLC previously treated with crizotinib (Table 1). All eight patients with crizotinib-naive *ALK*+ NSCLC had confirmed objective responses (confirmed ORR: 100% [95% CI: 63–100]; three patients had CRs and five patients had PRs), with median duration of response of 32.4 months (95% CI: 5.6–60.3).

**Table 1:** Investigator-Assessed Response Rates, PFS, and Overall Survival in the Phase 1/2 Study

Efficacy Parameter	Patients With *ALK*+ NSCLC			Patients With *ALK*+ NSCLC With Previous Crizotinib		
	All Doses (n = 79)	90 mg→180 mg Once Daily^a^ (n = 28)	180 mg Once Daily^b^ (n = 25)	All Doses (n = 71)	90 mg→180 mg Once Daily^a^ (n = 25)	180 mg Once Daily^b^ (n = 23)
Response characteristics						
Confirmed ORR, n (%)	53 (67)	22 (79)	17 (68)	45 (63)	19 (76)	15 (65)
[95% CI]	[56–77]	[59–92]	[47–85]	[51–75]	[55–91]	[43–84]
Confirmed CR, n (%)	8 (10)	4 (14)	2 (8)	5 (7)	3 (12)	2 (9)
Confirmed PR, n (%)	45 (57)	18 (64)	15 (60)	40 (56)	16 (64)	13 (57)
DCR, n (%)	70 (89)	25 (89)	20 (80)	62 (87)	22 (88)	18 (78)
[95% CI]	[80–95]	[72–98]	[59–93]	[77–94]	[69–98]	[56–93]
Time to response, median (range), mo	(n = 53) 1.9 (1.2–29.4)	(n = 22) 1.9 (1.2–6.0)	(n = 17) 1.9 (1.6–29.4)	(n = 45) 1.8 (1.2–29.4)	(n = 19) 1.8 (1.2–6.0)	(n = 15) 1.9 (1.6–29.4)
Duration of response, median (95% CI),^c^ mo	14.9 (9.9–29.5)	14.8 (7.9–33.3)	20.4 (7.6–44.5)	14.5 (9.0–22.1)	14.8 (7.9–25.1)	20.4 (7.5–51.6)
PFS						
No. of patients with events (%)	61 (77)	19 (68)	21 (84)	55 (77)	17 (68)	19 (83)
Median (95% CI),^c^ mo	14.5 (10.8–21.2)	16.3 (9.2–27.5)	14.5 (5.4–34.2)	13.4 (9.2–16.7)	14.7 (9.2–27.1)	14.5 (5.4–34.1)
PFS probability,^c^ % (95% CI)						
1 y	57 (45–68)	65 (43–80)	54 (32–71)	55 (42–66)	65 (42–81)	54 (32–72)
2 y	36 (25–47)	36 (17–56)	40 (21–59)	31 (20–43)	33 (14–54)	40 (19–59)
3 y	21 (12–32)	18 (5–38)	27 (11–46)	19 (10–29)	13 (2–34)	30 (12–49)
4 y	16 (8–26)	18 (5–38)	18 (6–36)	12 (5–23)	13 (2–34)	20 (6–39)
5 y	12 (5–22)	9 (1–31)	13 (3–30)	10 (4–20)	13 (2–34)	15 (4–33)
Overall survival						
No. of patients with events (%)	39 (49)	15 (54)	11 (44)	39 (54)	15 (60)	11 (48)
Median (95% CI),^c^ mo	47.6 (28.6–NR)	30.1 (22.5–NR)	55.0 (17.6–NR)	30.1 (21.4–55.0)	29.5 (21.4–NR)	51.2 (17.5–NR)
Overall survival probability,^c^ % (95% CI)						
1 y	79 (69–87)	86 (66–94)	79 (56–80)	77 (65–85)	84 (63–94)	76 (52–90)
2 y	65 (53–74)	68 (47–82)	69 (46–84)	61 (48–71)	64 (42–79)	66 (42–82)
3 y	52 (39–63)	42 (23–61)	64 (41–80)	46 (34–58)	37 (18–56)	61 (37–78)
4 y	47 (35–59)	42 (23–61)	58 (34–76)	41 (28–54)	37 (18–56)	54 (30–74)
5 y	42 (30–55)	42 (23–61)	43 (20–65)	35 (22–49)	37 (18–56)	39 (16–61)

CI, confidence interval; CR, complete response; DCR, disease control rate; NR, not reached; ORR, objective response rate; PFS, progression-free survival; PR, partial response.

a 180 mg once daily with 7-day lead-in at 90 mg.

b 90 mg twice daily or 180 mg once daily.

c Kaplan-Meier estimates of duration of response, PFS, and overall survival.

#### Progression-Free Survival

**Figure 2:**
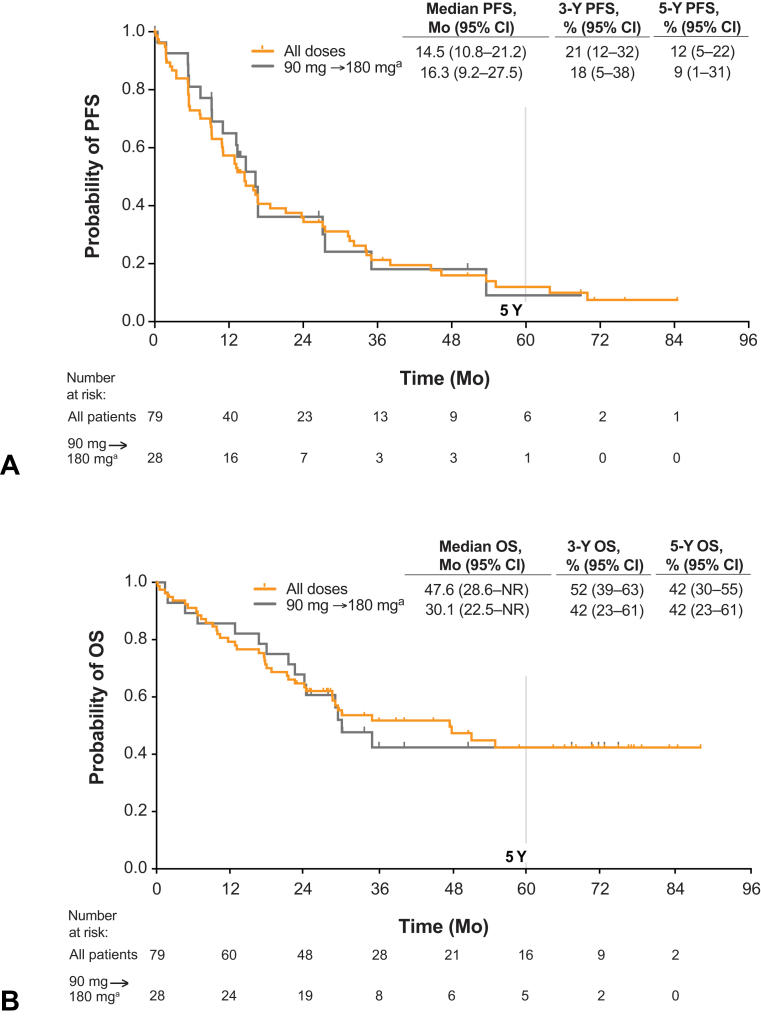
Efficacy of brigatinib in patients with *ALK*+ NSCLC in the phase 1/2 study. (*A*) Kaplan-Meier estimates of investigator-assessed PFS. Of the 79 patients with *ALK*+ NSCLC, 61 (77%) had an event. (*B*) OS. Of the 79 patients, 39 (49%) died. Tick marks in Kaplan-Maier plots indicate censored data. ^a^180 mg once daily with 7-day lead-in at 90 mg. *ALK*+, *ALK* rearrangement positive; CI, confidence interval; NR, not reached; OS, overall survival; PFS, progression-free survival.

For the 79 patients with *ALK*+ NSCLC, the KM-estimated median PFS was 14.5 months (95% CI: 10.8–21.2), with PFS rates of 21% (95% CI: 12–32) at 3 years and 12% (95% CI: 5–22) at 5 years (Fig. 2*A*; Table 1). In the 28 patients with *ALK*+ NSCLC treated at 180 mg once daily with 7-day lead-in at 90 mg, median PFS was 16.3 months (95% CI: 9.2–27.5), with PFS rates of 18% (95% CI: 5–38) at 3 years and 9% (95% CI: 1–31) at 5 years (Table 1). For the 71 patients with crizotinib-pretreated *ALK*+ NSCLC, the KM-estimated median PFS was 13.4 months (95% CI: 9.2–16.7), with event-free rates of 19% (95% CI: 10–29) at 3 years and 10% (95% CI: 4–20) at 5 years (Table 1). For the 25 patients with crizotinib-pretreated *ALK*+ NSCLC treated at 180 mg once daily with 7-day lead-in at 90 mg, median PFS was 14.7 months (95% CI: 9.2–27.1), with PFS rates of 13% (95% CI: 2–34) at 3 years and 13% (95% CI: 2–34) at 5 years (Table 1). Among the eight patients with crizotinib-naive *ALK*+ NSCLC, median PFS was 34.2 months (95% CI: 7.4–63.9), with PFS rates of 45% (95% CI: 11–75) at 3 years and 30% (95% CI: 4–63) at 5 years.

Intracranial response and PFS data were not collected consistently in the phase 1/2 study and therefore could not be analyzed.

#### Overall Survival

For the 79 patients with *ALK*+ NSCLC, KM-estimated median overall survival was 47.6 months (95% CI: 28.6–not reached [NR]) and probability of survival at 5 years was 42% (95% CI: 30–55; Fig. 2*B* and Table 1). In the 71 patients with crizotinib-pretreated *ALK*+ NSCLC, median overall survival was 30.1 months (95% CI: 21.4–55.0), and 5-year overall survival probability was 35% (95% CI: 22–49). All eight patients with crizotinib-naive *ALK*+ NSCLC were alive 2 years after the first dose (protocol-specified follow-up period for overall survival).

### Efficacy: ALTA

#### Overall Efficacy

**Figure 3:**
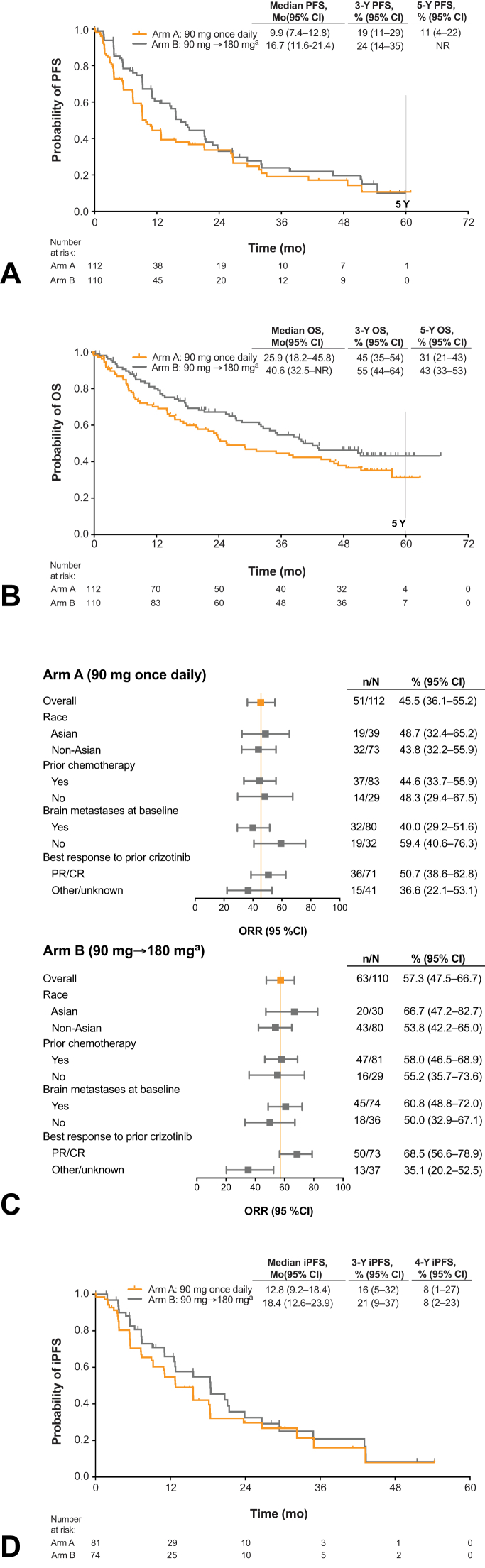
Brigatinib efficacy in patients with crizotinib-refractory *ALK*+ NSCLC in ALTA. (*A*) Kaplan-Maier estimates of IRC-assessed PFS in the ITT population. Of the 112 patients in arm A, 73 (65%) had an event; of the 110 patients in arm B, 62 (56%) had an event. (*B*) OS. Of the 112 patients in arm A, 64 (57%) died; of the 110 patients in arm B, 54 (49%) died. (*C*) Forest plot of subgroup analyses of investigator-assessed confirmed ORR. (*D*) Intracranial PFS in patients with any brain metastases (measurable or nonmeasurable) per the IRC at baseline. Of the 81 assessable patients in arm A, 43 (53%) had an event; of the 74 assessable patients in arm B, 35 (47%) had an event. Tick marks in Kaplan-Maier plots indicate censored data. ^a^180 mg once daily with 7-day lead-in at 90 mg. *ALK*+, *ALK* rearrangement positive; CR, complete response; iPFS, intracranial progression-free survival; IRC, independent review committee; ITT, intention-to-treat; NR, not reached; ORR, objective response rate; OS, overall survival; PFS, progression-free survival; PR, partial response.

In the final analysis of ALTA, the confirmed ORR per investigator assessment was 46% (97.5% CI: 35–57) in arm A and 57% (97.5% CI: 46–68) in arm B, with median duration of response of 12.0 months (95% CI: 9.2–19.4) and 13.8 months (95% CI: 10.8–17.6), respectively (Table 2). The confirmed ORR per IRC assessment was 52% (95% CI: 42–61) in arm A and 56% (95% CI: 47–66) in arm B. Median investigator-assessed PFS was 9.2 months (95% CI: 7.4–11.1) in arm A and 15.6 months (95% CI: 11.1–18.5) in arm B. The investigator-assessed PFS rate at 3 years was 15% (95% CI: 8–23) in arm A and 18% (95% CI: 10–27) in arm B and at 4 years was 9% (95% CI: 4–18) and 15% (95% CI: 8–24), respectively (Table 2). Median IRC-assessed PFS was 9.9 months (95% CI: 7.4–12.8) in arm A and 16.7 months (95% CI: 11.6–21.4) in arm B (Fig. 3*A*), with event-free rates of 19% (95% CI: 11–29) in arm A and 24% (95% CI: 14–35) in arm B at 3 years and 17% (95% CI: 9–27) in arm A and 20% (95% CI: 11–31) in arm B at 4 years (Table 2).

**Table 2:** Objective Responses Rates, PFS, and Overall Survival in ALTA

Efficacy Parameter	Investigator-Assessed		IRC-Assessed	
	Arm A 90 mg Once Daily (n = 112)	Arm B 90 mg→180 mg Once Daily^a^ (n = 110)	Arm A 90 mg Once Daily (n = 112)	Arm B 90 mg→180 mg Once Daily^a^ (n = 110)
All patients				
Confirmed ORR, n (%)	51 (46)	63 (57)	58 (52)	62 (56)
[97.5% CI]^b^ or [95% CI]	[35–57]^b^	[46–68]^b^	[42–61]	[47–66]
Confirmed CR, n (%)	2 (2)	5 (5)	7 (6)	8 (7)
Confirmed PR, n (%)	49 (44)	58 (53)	51 (46)	54 (49)
DCR, n (%)	91 (81)	95 (86)	87 (78)	92 (84)
[95% CI]	[73–88]	[79–92]	[69–85]	[75–90]
Time to response, median (range), mo	(n = 51) 1.8 (1.7–11.1)	(n = 63) 1.9 (1.0–35.0)	(n = 58) 1.8 (1.6–37.8)	(n = 62) 1.9 (1.0–23.4)
Duration of response, median (95% CI),^c^ mo	12.0 (9.2–19.4)	13.8 (10.8–17.6)	19.4 (9.2–24.9)	15.7 (13.6–22.1)
PFS				
No. of patients with events (%)	85 (76)	72 (65)	73 (65)	62 (56)
Median (95% CI),^c^ mo	9.2 (7.4–11.1)	15.6 (11.1–18.5)	9.9 (7.4–12.8)	16.7 (11.6–21.4)
PFS probability,^c^ % (95% CI)				
1 y	37 (27–46)	58 (47–67)	44 (34–54)	61 (49–70)
2 y	23 (15–32)	31 (22–42)	34 (24–44)	33 (22–44)
3 y	15 (8–23)	18 (10–27)	19 (11–29)	24 (14–35)
4 y	9 (4–18)	15 (8–24)	17 (9–27)	20 (11–31)
5 y	NR	NR	11 (4–22)	NR
Overall survival	Arm A (n = 112)		Arm B (n = 110)	
No. of patients with events (%)	64 (57)		54 (49)	
Median (95% CI),^c^ mo	25.9 (18.2–45.8)		40.6 (32.5–NR)	
Overall survival probability,^c^ % (95% CI)				
1 y	70 (60–78)		80 (71–87)	
2 y	55 (44–64)		67 (57–75)	
3 y	45 (35–54)		55 (44–64)	
4 y	38 (28–48)		46 (36–56)	
5 y	31 (21–43)		43 (33–53)	

CI, confidence interval; CR, complete response; DCR, disease control rate; IRC, independent review committee; NR, not reached; ORR, objective response rate; PFS, progression-free survival; PR, partial response.

a 180 mg once daily with 7-day lead-in at 90 mg.

b Primary end point tested at 0.025 alpha level for each dose.

c Kaplan-Meier estimates of duration of response.

Median overall survival was 25.9 months (95% CI: 18.2–45.8) in arm A and 40.6 months (32.5–NR) in arm B (Fig. 3*B*). Probability of survival at 5 years was 31% in arm A and 43% in arm B (Table 2 and Fig. 3*B*).

Exploratory subgroup analyses were performed for confirmed ORR (Fig. 3*C*), PFS (Supplementary Table 1), and overall survival (Supplementary Table 2) by race (Asian and non-Asian), previous chemotherapy, brain metastases at baseline, and best response with previous crizotinib therapy. There were no notable differences in any of these efficacy parameters between subgroups or when compared with the overall ITT population.

#### Intracranial Efficacy

The IRC-assessed intracranial confirmed ORR in patients with measurable brain metastases at baseline was 50% (13 of 26; 95% CI: 30–70) in arm A and 67% (12 of 18; 95% CI: 41–87) in arm B (Supplementary Table 3). KM-estimated median duration of intracranial response was 9.4 months (95% CI: 3.7–NR) in arm A and 16.6 months (95% CI: 3.7–NR) in arm B.

KM-estimated median intracranial PFS for patients with any brain metastases at baseline was 12.8 months (95% CI: 9.2–18.4) in arm A and 18.4 months (95% CI: 12.6–23.9) in arm B (Fig. 3*D*). Median intracranial PFS in patients with measurable brain metastases at baseline was 11.1 months (95% CI: 5.6–26.7) in arm A and 18.5 months (95% CI: 4.9–NR) in arm B.

In patients with brain metastases at baseline, median overall survival was 29.5 months (95% CI: 15.9–51.7) in arm A and 51.1 months (95% CI: 34.1–NR) in arm B; for patients without brain metastases at baseline, median overall survival was 24.1 months (95% CI: 9.2–48.9) in arm A and 32.5 months (95% CI: 17.9–NR) in arm B.

### Safety

With long-term follow-up, no new safety signals were identified compared with previous analyses.^15^, ^16^, ^17^ Treatment-related AEs reported in more than 10% of patients and grade 3 or greater treatment-related AEs reported in more than 3% of patients are listed in Supplementary Table 4. The median dose intensity was 174 mg/d in the 79 patients with *ALK*+ NSCLC in the phase 1/2 study, 90 mg/d in ALTA arm A, and 169 mg/d in ALTA arm B. Dose reduction because of any AE occurred in 13% (10 of 79) of patients in the phase 1/2 study, 8% (9 of 109) of treated patients in ALTA arm A, and 33% (36 of 110) of treated patients in ALTA arm B. Among patients with *ALK*+ NSCLC in the phase 1/2 study, median time to dose reduction (for any reason) was 37 days in one of 14 patients with dose reduction from a starting dose of 90 mg once daily, 28 days (range: 11–29) in three of six patients at 120 mg/d, 86 days (23–1491) in 11 of 28 at 180 mg once daily with 7-day lead-in at 90 mg, 304 days (21–1345) in seven of 25 patients at 180 mg/d, and 34 days in one of five patients at ≥240 mg/d. In ALTA, the median time to dose reduction was 27 days (range: 1–288) for 10 of 109 patients with dose reduction in arm A and 138 days (range: 8–1195) for 41 of 110 patients in arm B. The most common AE leading to dose reduction was increased lipase level (5%) in the phase 1/2 study and increased blood creatine phosphokinase level in ALTA (2% in arm A and 9% in arm B; Supplementary Table 5). Dose interruption because of any AE occurred in 59% (47 of 79) of patients in the phase 1/2 study and 49% (53 of 109) and 61% (67 of 110) of treated patients in ALTA arms A and B, respectively. Discontinuation because of any AE occurred in 10% (8 of 79) of patients in the phase 1/2 study and 4% (4 of 109) and 13% (14 of 110) of treated patients in ALTA arms A and B, respectively. Rates of interstitial lung disease and pneumonitis in both studies were similar to previous reports with longer follow-up.^15^, ^16^, ^17^

In the phase 1/2 study, 15 of the 79 patients with *ALK*+ NSCLC died within 30 days of the last dose of brigatinib; two deaths were found to be possibly related to brigatinib (unexpected death on day 568 in a patient receiving 90 mg once daily and sepsis on day 541 in a patient allocated to 180 mg once daily with 7-day lead-in at 90 mg). In ALTA, 36 patients (22 in arm A and 14 in arm B) died within 30 days of the last dose of brigatinib; one death was found to be possibly related to brigatinib treatment (sudden death on day 3 in a patient in arm B).

## Discussion

In the final analysis of the phase 1/2 study, brigatinib was found to have sustained long-term activity and PFS in patients with *ALK*+ NSCLC at a median follow-up of 27.7 months (range: 0.2–88.3) and more than 5 years after the last patient was enrolled. In an earlier report of results from the phase 1/2 study, brigatinib had encouraging CNS activity, with favorable intracranial objective responses and PFS at total daily doses of 90 mg or greater.

The sustained long-term activity of brigatinib in patients with crizotinib-refractory *ALK*+ NSCLC was confirmed in the final analysis of ALTA at a median follow-up of 19.6 months (range: 0.1–62.8) in arm A and 28.3 months (range: 0.1–66.8) in arm B, and more than 4 years after the last patient was enrolled. The approved dosing regimen (180 mg once daily with 7-d lead-in at 90 mg; arm B) was associated with numerically higher ORR, PFS, and overall survival than the 90 mg daily dose (arm A).

Brigatinib also exhibited sustained intracranial activity in patients with baseline brain metastases. It seems that patients with brain metastases at baseline had better median overall survival than patients without brain metastases. Nevertheless, PFS rates of these two subgroups do not reveal the same trend. If poststudy treatments are not considered and if brain metastasis is considered as the primary form of ALK TKI failure, these results may not seem as intriguing. One potential explanation is that patients with brain metastases at baseline may seem to have better median overall survival because they were treated with brigatinib despite having confirmed brain metastasis, whereas patients without brain metastases at baseline would have discontinued brigatinib on intracranial disease progression. It is possible that without brigatinib protection, death may occur sooner after intracranial progression.

Brigatinib seems to compare favorably with other TKIs in the second-line setting. In patients with crizotinib-pretreated *ALK*+ NSCLC, alectinib has an IRC-assessed ORR of 51%, median duration of response of 14.9 months, median PFS of 8.3 months,^19^ and median overall survival of 29.1 months.^20^ Alectinib was associated with an intracranial ORR (by IRC) of 64%, with median duration of intracranial response of 10.8 months, in patients with measurable brain metastases at baseline (by RECIST version 1.1).^21^^,^^22^ Similarly, ceritinib has an ORR of 39% to 43% (by investigator assessment), median duration of response of 6.9 to 9.7 months, median PFS (by investigator assessment) of 5.7 to 6.7 months, and median overall survival of 14.9 months in patients with *ALK*+ NSCLC previously treated with chemotherapy and crizotinib^6^^,^^23^; among patients with measurable brain metastases, the intracranial ORR was 35%, with median duration of intracranial response of 6.9 months.^6^ Lorlatinib has numerically higher overall (ORR: 73%) and intracranial (70%) response rates in crizotinib-pretreated patients, although median PFS (11.1 mo)^24^ seems to be shorter than that observed with brigatinib (16.7 mo) and mature overall survival data are not yet available for this setting.

Crizotinib was the first ALK inhibitor to obtain FDA approval for use in patients with treatment-naive *ALK*+ NSCLC.^25^^,^^26^ Second- and third-generation ALK TKIs (alectinib, brigatinib, ceritinib, and lorlatinib) have efficacy in the treatment of patients with ALK TKI-naive *ALK+* NSCLC and have replaced crizotinib as recommended first-line treatments for patients with *ALK*+ NSCLC.^11^^,^^27^, ^28^, ^29^, ^30^, ^31^ Optimal sequencing of next-generation TKIs in TKI-refractory *ALK*+ NSCLC has not been established. The phase 2 J-ALTA trial assessed the efficacy of brigatinib in 47 Japanese patients with advanced *ALK*+ NSCLC refractory to alectinib, with or without previous use of crizotinib.^32^ Brigatinib had clinically meaningful efficacy, with an ORR (by IRC) of 34%, median duration of response of 11.8 months, and median PFS (by IRC) of 7.3 months.^32^ A multinational phase 2 trial (ALTA-2, NCT03535740) has enrolled 104 patients to investigate brigatinib efficacy and safety in patients with *ALK*+ NSCLC in the post-alectinib or post-ceritinib setting.^33^

The safety profile of brigatinib was consistent with previous reports, with no new safety concerns noted.^15^, ^16^, ^17^ The most common AEs were gastrointestinal events and elevated blood creatine phosphokinase levels. There were no changes in the incidence of pulmonary AEs with early onset because results were reported in previous publications.^15^, ^16^, ^17^ In ALTA, dose reductions were more common at the phase 2 recommended dose of 180 mg once daily after a 7-day lead-in at 90 mg, but these did not seem to compromise efficacy.

In conclusion, brigatinib had sustained long-term activity, PFS, and manageable safety in patients with *ALK*+ NSCLC. The 180 mg daily dose after 7-day lead-in at 90 mg was associated with numerically longer median PFS and overall survival than the 90-mg daily dose. Final efficacy results of the phase 1/2 and phase 2 (ALTA) trials of brigatinib are similar, if not superior, to those reported for other approved ALK TKIs in the second-line setting. These data and the prospect of prolonged survival in this setting cement the role of next-generation ALK TKIs such as brigatinib in the treatment of patients with advanced *ALK+* NSCLC.

## CRediT Authorship Contribution Statement

**Scott N. Gettinger, Rudolf M. Huber, Corey J. Langer, Edward S. Kim, Harry J. M. Groen:** Conceptualization.

**Scott N. Gettinger, Rudolf M. Huber, Lyudmila Bazhenova, Karen L. Reckamp, Glen J. Weiss:** Data curation.

**Scott N. Gettinger, Corey J. Langer, Joanna Pye, Yuyin Liu, Pingkuan Zhang, Florin Vranceanu:** Formal analysis.

**Scott N. Gettinger, Rudolf M. Huber, Dong-Wan Kim, Lyudmila Bazhenova, Karin Holmskov Hansen, Marcello Tiseo, Corey J. Langer, Luis G. Paz-Ares Rodríguez, Howard L. West, Karen L. Reckamp, Glen J. Weiss, Egbert F. Smit, Maximilian J. Hochmair, Sang-We Kim, Myung-Ju Ahn, Edward S. Kim, Harry J.M. Groen, D. Ross Camidge:** Investigation.

**Scott N. Gettinger, Corey J. Langer, D. Ross Camidge:** Methodology.

**Scott N. Gettinger, Lyudmila Bazhenova, Corey J. Langer, D. Ross Camidge:** Project administration.

**Scott N. Gettinger, Corey J. Langer, Luis G. Paz-Ares Rodríguez, Karen L. Reckamp, D. Ross Camidge:** Resources.

**Scott N. Gettinger, Luis G. Paz-Ares Rodríguez, Karen L. Reckamp. Glen J. Weiss, Egbert F. Smit, Harry J. M. Groen, D. Ross Camidge:** Supervision.

**Rudolf M. Huber, Marcello Tiseo:** Validation.

**Marcello Tiseo, Edward S. Kim:** Visualization.

**Scott N. Gettinger, Rudolf M. Huber, Dong-Wan Kim, Lyudmila Bazhenova, Karin Holmskov Hansen, Marcello Tiseo, Corey J. Langer, Luis G. Paz-Ares Rodríguez, Howard L. West, Karen L. Reckamp, Glen J. Weiss, Egbert F. Smit, Maximilian J. Hochmair, Sang-We Kim, Myung-Ju Ahn, Edward S. Kim, Harry J.M. Groen, Joanna Pye, Yuyin Liu, Pingkuan Zhang, Florin Vranceanu, D. Ross Camidge:** Writing—original draft; Writing—review and editing.
