# Supplementary material for: Long-Term Efficacy and Safety of Brigatinib in Crizotinib-Refractory ALK+ NSCLC: Final Results of the Phase 1/2 and Randomized Phase 2 (ALTA) Trials
Source: JTO Clin Res Rep. 2022 Jul 31;3(9):100385. doi: 10.1016/j.jtocrr.2022.100385 (PMC9440305; doi:10.1016/j.jtocrr.2022.100385)
Supplement: Supplementary material [file mmc1.docx]

**SUPPLEMENTAL DATA**

**Table S1.** Subgroup Analyses of Investigator-Assessed PFS in ALTA

|  | **Arm A: 90 mg qd (n=112)** | | | | **Arm B: 90 mg→180 mg qd^a^ (n=110)** | | | |
| --- | --- | --- | --- | --- | --- | --- | --- | --- |
| **Subgroup** | **N** | **Events,**  **n (%)** | **Median PFS**  **(95% CI), mo** | **HR (95% CI)** | **N** | **Events,**  **n (%)** | **Median PFS**  **(95% CI), mo** | **HR (95% CI)** |
| Race |  |  |  |  |  |  |  |  |
| Asian | 39 | 33 (85) | 9.2 (5.6–15.6) | Reference | 30 | 20 (67) | 15.6 (11.1–15.7) | Reference |
| Non-Asian | 73 | 52 (71) | 9.2 (5.7–11.1) | 0.92 (0.59–1.42) | 80 | 52 (65) | 14.7 (10.8–22.8) | 0.97 (0.58–1.62) |
| Prior chemotherapy |  |  |  |  |  |  |  |  |
| Yes | 83 | 64 (77) | 8.2 (5.6–11.1) | Reference | 81 | 50 (62) | 15.6 (11.1–22.3) | Reference |
| No | 29 | 21 (72) | 9.3 (7.4–21.0) | 0.86 (0.53–1.41) | 29 | 22 (76) | 12.6 (4.9–15.7) | 1.50 (0.91–2.48) |
| Brain metastases at baseline |  |  |  |  |  |  |  |  |
| Yes | 80 | 60 (75) | 9.3 (7.3–11.1) | Reference | 74 | 53 (72) | 15.6 (10.8–21.1) | Reference |
| No | 32 | 25 (78) | 7.4 (5.6–11.3) | 0.99 (0.62–1.59) | 36 | 19 (53) | 15.6 (11.1–23.7) | 0.85 (0.50–1.44) |
| Best response to prior crizotinib |  |  |  |  |  |  |  |  |
| PR or CR | 71 | 52 (73) | 11.0 (7.4–18.2) | Reference | 73 | 46 (63) | 15.6 (11.1–21.1) | Reference |
| Other or unknown | 41 | 33 (80) | 7.4 (3.7–9.3) | 1.58 (1.02–2.45) | 37 | 26 (70) | 12.9 (5.2–21.1) | 1.50 (0.92–2.43) |
| ^a^ 180 mg qd with 7-day lead-in at 90 mg  CR, complete response; HR, hazard ratio; PFS, progression-free survival; PR, partial response; qd, once daily | | | | | | | | |

**Table S2.** Subgroup Analyses of Overall Survival in ALTA

|  | **Arm A: 90 mg qd (n=112)** | | | | **Arm B: 90 mg→180 mg qd^a^ (n=110)** | | | |
| --- | --- | --- | --- | --- | --- | --- | --- | --- |
| **Subgroup** | **N** | **Events,**  **n (%)** | **Median OS**  **(95% CI), mo** | **HR (95% CI)** | **N** | **Events,**  **n (%)** | **Median OS**  **(95% CI), mo** | **HR (95% CI)** |
| Race |  |  |  |  |  |  |  |  |
| Asian | 39 | 19 (49) | 29.3 (18.2–NR) | Reference | 30 | 12 (40) | 40.2 (28.8–NR) | Reference |
| Non-Asian | 73 | 45 (62) | 25.8 (14.5–45.8) | 1.17 (0.68–2.00) | 80 | 42 (53) | 42.4 (25.7–NR) | 1.24 (0.65–2.36) |
| Prior chemotherapy |  |  |  |  |  |  |  |  |
| Yes | 83 | 55 (66) | 22.3 (14.4–37.9) | Reference | 81 | 37 (46) | 51.1 (35.3–NR) | Reference |
| No | 29 | 9 (31) | 57.7 (24.3–57.7) | 0.42 (0.21–0.85) | 29 | 17 (59) | 21.6 (10.1–NR) | 1.73 (0.97–3.08) |
| Brain metastases at baseline |  |  |  |  |  |  |  |  |
| Yes | 80 | 43 (54) | 29.5 (15.9–51.7) | Reference | 74 | 35 (47) | 51.1 (34.1–NR) | Reference |
| No | 32 | 21 (66) | 24.1 (9.2–48.9) | 1.16 (0.69–1.95) | 36 | 19 (53) | 32.5 (17.9–NR) | 1.39 (0.80–2.44) |
| Best response to prior crizotinib |  |  |  |  |  |  |  |  |
| PR or CR | 71 | 34 (48) | 37.9 (22.3–NR) | Reference | 73 | 38 (52) | 40.2 (28.8–NR) | Reference |
| Other or unknown | 41 | 30 (73) | 20.2 (10.6–24.6) | 1.98 (1.21–3.25) | 37 | 16 (43) | NR (13.0–NR) | 0.94 (0.53–1.69) |

^a^ 180 mg qd with 7-day lead-in at 90 mg

CR, complete response; HR, hazard ratio; NR, not reached; OS, overall survival; PR, partial response; qd, once daily

**Table S3.** Intracranial Response Rates in Patients with Measurable CNS Metastases at Baseline Per IRC in ALTA

|  | **IRC-Assessed** | |
| --- | --- | --- |
|  | **Arm A**  **90 mg qd**  **(n=26)** | **Arm B**  **90 mg→180 mg qd^a^**  **(n=18)** |
| Confirmed intracranial ORR, n (%) | 13 (50) | 12 (67) |
| [95% CI] | [30–70] | [41–87] |
| Confirmed intracranial CR, n (%) | 2 (8) | 0 |
| Confirmed intracranial PR, n (%) | 11 (42) | 12 (67) |
| Intracranial DCR, n (%) | 22 (85) | 15 (83) |
| [95% CI] | [65–96] | [59–96] |
| Duration of intracranial response in responders, median (95% CI),^b^ months | (n=13)  9.4 (3.7–NR) | (n=12)  16.6 (3.7–NR) |

^a^ 180 mg qd with 7-day lead-in at 90 mg

^b^ Duration of CNS response is defined as time of first CR or PR in the CNS among confirmed responders until progression or death

CR, complete response; CNS, central nervous system; DCR, disease control rate; IRC, independent review committee; NR, not reached; ORR, objective response rate; PR, partial response; qd, once daily

**Table S4.** Treatment-Emergent Adverse Events of Any Grade Reported in >10% of Patients or Grade ≥3 Reported in >3% of Patients

|  | **Phase 1/2 Study**  **Patients With *ALK*+ NSCLC (n=79)** | | **ALTA**  **Arm A: 90 mg qd**  **(n=109)** | | **ALTA**  **Arm B: 90 mg→180 mg qd^a^**  **(n=110)** | |
| --- | --- | --- | --- | --- | --- | --- |
| **Adverse Event** | **Any Grade** | **Grade ≥3** | **Any Grade** | **Grade ≥3** | **Any Grade** | **Grade ≥3** |
| Diarrhea | 38 (48) | 1 (1) | 19 (17) | 0 | 39 (35) | 0 |
| Nausea | 40 (51) | 1 (1) | 28 (26) | 0 | 37 (34) | 1 (1) |
| Increased blood creatine phosphokinase^b^ | 13 (16) | 4 (5) | 19 (17) | 7 (6) | 37 (34) | 15 (14) |
| Fatigue | 31 (39) | 2 (3) | 13 (12) | 1 (1) | 22 (20) | 0 |
| Vomiting | 16 (20) | 0 | 16 (15) | 0 | 21 (19) | 0 |
| Muscle spasms | 15 (19) | 0 | 10 (9) | 0 | 21 (19) | 0 |
| Increased aspartate aminotransferase | 22 (28) | 2 (3) | 12 (11) | 0 | 20 (18) | 3 (3) |
| Increased lipase^c^ | 24 (30) | 14 (18) | 12 (11) | 6 (6) | 20 (18) | 9 (8) |
| Hypertension | 13 (16) | 6 (8) | 8 (7) | 5 (5) | 20 (18) | 6 (6) |
| Increased amylase^c^ | 21 (27) | 4 (5) | 13 (12) | 2 (2) | 18 (16) | 2 (2) |
| Increased alanine aminotransferase | 14 (18) | 2 (3) | 12 (11) | 0 | 14 (13) | 4 (4) |
| Cough | 12 (15) | 1 (1) | 3 (3) | 0 | 11 (10) | 0 |
| Dyspnea | 12 (15) | 2 (3) | 8 (7) | 0 | 8 (7) | 0 |
| Constipation | 10 (13) | 0 | 5 (5) | 0 | 8 (7) | 0 |
| Dry mouth | 9 (11) | 0 | 3 (3) | 0 | 7 (6) | 0 |
| Increased blood insulin | 12 (15) | 0 | 0 | 0 | 1 (1) | 0 |
| Hypophosphatemia | 12 (15) | 4 (5) | 0 | 0 | 1 (1) | 1 (1) |

^a^ 180 mg qd with 7-day lead-in at 90 mg

^b^ Neither myalgia nor musculoskeletal pain of any grade were reported in any patients

^c^ No clinical cases of pancreatitis were reported

*ALK*+, anaplastic lymphoma kinase rearrangement–positive; NSCLC, non–small cell lung cancer; qd, once daily

**Table S5.** Adverse Events Leading to Dose Reduction in ≥2 Patients in the Phase 1/2 Study or the ALTA Trial

|  | **Phase 1/2 Study**  **Patients With *ALK*+ NSCLC**  **(n=79)** | **ALTA**  **Arm A: 90 mg qd^a^**  **(n=109)** | **ALTA**  **Arm B: 90 mg→180 mg qd^b^**  **(n=110)** |
| --- | --- | --- | --- |
| **Patients with ≥1 AE leading to dose reduction, n (%)** | 10 (13) | 9 (8) | 36 (33) |
| Increased blood creatine phosphokinase | 0 | 2 (2) | 10 (9) |
| Increased lipase | 4 (5) | 1 (1) | 5 (5) |
| Pneumonitis | 0 | 1 (1) | 3 (3) |
| Increased amylase | 2 (3) | 1 (1) | 2 (2) |
| Nausea | 2 (3) | 0 | 2 (2) |
| Decreased appetite | 0 | 0 | 2 (2) |
| Electrocardiogram QT interval prolonged | 0 | 0 | 2 (2) |
| Hyponatremia | 0 | 0 | 2 (2) |
| Rash, maculopapular | 0 | 0 | 2 (2) |
| Cough | 0 | 1 (1) | 1 (1) |
| Hypertension | 0 | 1 (1) | 1 (1) |
| Rash, erythematous | 0 | 1 (1) | 1 (1) |

^a^ For arm A, dose modification was required for any grade 3 or 4 nonhematologic toxicity, including laboratory abnormalities.

- - - - Grade 3: For 90 mg once daily dose, hold until event is grade 1 or lower, or has returned to baseline. Resume at 90 mg once daily or 60 mg once daily (at investigator’s discretion). For recurrence at 90 mg once daily, hold until event is grade 1 or lower, or has returned to baseline, and resume treatment at 60 mg once daily. When the current dose is 60 mg once daily, consider discontinuing treatment
- Grade 4: For 90 mg once-daily dose, hold until event is grade 1 or lower, or has returned to baseline. Resume treatment at 60 mg once daily or discontinue (at investigator’s discretion). When the current dose is 60 mg once daily, consider discontinuing treatment

^b^ For arm B, dose modification was required for grade 2 events lasting longer than 3 days or any grades 3 or 4 nonhematologic toxicity, includinglaboratory abnormalities

- For 90 mg once daily dose (previous to dose escalation):
- Grade 2 (>3 days) and grade 3: Hold until event is grade 1 or lower, or has returned to baseline. Resume at 90 mg once daily (at investigator’s discretion)
- Grade 4: Hold until event is grade 1 or lower, or has returned to baseline. Resume treatment at 60 mg once daily or discontinue (at investigator’s discretion)
- After dose escalation:
- Grade 3: When the dose is 180 mg once daily, hold until event is grade 1 or lower, or has returned to baseline and then resume at 180 mg once daily or 120 mg once daily (at investigator’s discretion). When the current dose is 120 mg once daily, hold until event is grade 1 or lower, or has returned to baseline and resume at 90 mg once daily after recovery. When the current dose is 90 mg once daily, hold until event is grade 1 or lower, or has returned to baseline and resume at 60 mg once daily after recovery, or discontinue (at investigator’s discretion). When the current dose is 60 mg once daily, consider discontinuing treatment
- Grade 4: When the current dose is 180 mg once daily, hold until event is grade 1 or lower, or has returned to baseline; resume at 120 mg once daily, or discontinue (at investigator’s discretion) When the current dose is 120 mg once daily, hold until event is grade 1 or lower, or has returned to baseline. Resume at 90 mg once daily or discontinue (at investigator’s discretion). When the current dose is 90 mg once daily, hold until event is grade 1 or lower, or has returned to baseline and resume at 60 mg once daily after recovery, or discontinue (at investigator’s discretion). When the current dose is 60 mg once daily, consider discontinuing treatment
